# 4-Hydr­oxy-3-mesityl-1-oxaspiro­[4.4]non-3-en-2-one

**DOI:** 10.1107/S1600536809008617

**Published:** 2009-03-14

**Authors:** Jin-Hao Zhao, Yong Zhou, Guo-Nian Zhu, Jing-Li Cheng

**Affiliations:** a College of Agriculture and Biotechnology, Zhejiang University, Hangzhou 310029, People’s Republic of China; b College of Chemical Engineering and Materials Science, Zhejiang University of Technology, Hangzhou 310032, People’s Republic of China

## Abstract

In the title compound, C_17_H_20_O_3_, the five-membered cyclo­pentyl ring displays an envelope conformation, with the atom at the flap position 0.538 (3) Å out of the mean plane formed by the other four atoms. The dihedral angle between the benzene and furan rings is 63.34 (15)°. In the crystal structure, mol­ecules are linked through inter­molecular O—H⋯O hydrogen bonds, forming a zigzag chain along [101].

## Related literature

For related compounds, see: Fischer *et al.* (1995 ▶); Bayer Aktiengesellschaft (1995 ▶). For a related structure, see: Yu *et al.* (2009 ▶).
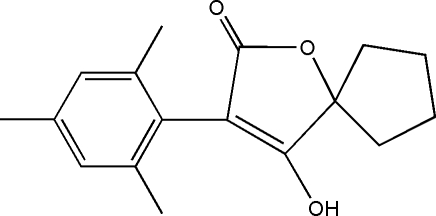


## Experimental

### 

#### Crystal data

C_17_H_20_O_3_
*M* *_r_* = 272.33
Monoclinic, 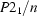

*a* = 8.8543 (4) Å
*b* = 17.9266 (7) Å
*c* = 9.4883 (4) Å
β = 97.809 (2)°
*V* = 1492.09 (11) Å^3^
*Z* = 4
Mo *K*α radiation
μ = 0.08 mm^−1^
*T* = 296 K
0.54 × 0.48 × 0.20 mm

#### Data collection

Rigaku R-AXIS RAPID diffractometer
Absorption correction: multi-scan (**ABSCOR**; Higashi, 1995 ▶) *T*_min_ = 0.947, *T*_max_ = 0.984
14502 measured reflections
3410 independent reflections
2344 reflections with *I* > 2σ(*I*)
*R*_int_ = 0.026

#### Refinement

*R*[*F* ^2^ > 2σ(*F* ^2^)] = 0.045
*wR*(*F* ^2^) = 0.139
*S* = 1.00
3410 reflections
186 parameters
H-atom parameters constrained
Δρ_max_ = 0.21 e Å^−3^
Δρ_min_ = −0.17 e Å^−3^

### 

Data collection: *PROCESS-AUTO* (Rigaku, 1998 ▶); cell refinement: *PROCESS-AUTO*; data reduction: *CrystalStructure* (Rigaku/MSC, 2002 ▶); program(s) used to solve structure: *SHELXS97* (Sheldrick, 2008 ▶); program(s) used to refine structure: *SHELXL97* (Sheldrick, 2008 ▶); molecular graphics: *ORTEP-3* (Farrugia, 1997 ▶); software used to prepare material for publication: *WinGX* (Farrugia, 1999 ▶).

## Supplementary Material

Crystal structure: contains datablocks global, I. DOI: 10.1107/S1600536809008617/is2389sup1.cif

Structure factors: contains datablocks I. DOI: 10.1107/S1600536809008617/is2389Isup2.hkl

Additional supplementary materials:  crystallographic information; 3D view; checkCIF report

## Figures and Tables

**Table 1:** Hydrogen-bond geometry (Å, °)

*D*—H⋯*A*	*D*—H	H⋯*A*	*D*⋯*A*	*D*—H⋯*A*
O1—H1⋯O2^i^	0.82	1.87	2.6267 (14)	154

Symmetry code: (i) 
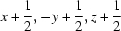
.

**Fig. 1.**
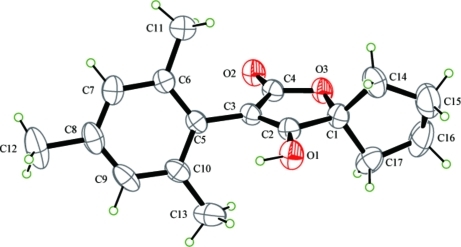
The molecular structure of (I), showing the atom-labelling scheme. Displacement ellipsoids are drawn at the 30% probability level.

**Fig. 2.**
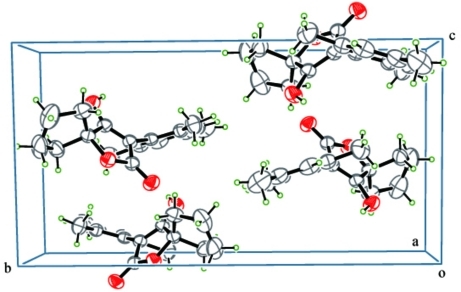
Molecular packing arrangement in the unit cell.

**Fig. 3.**
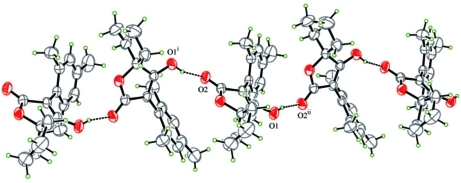
View showing the O—H···O hydrogen bonding (dashed lines). [Symmetry codes: (i) -1/2 + x, 1/2 - y, -1/2 + z; (ii) 1/2 + x, 1/2 - y, 1/2 + z.]

## supplementary crystallographic information

### Comment

Substituted 4-hydroxy-1-oxaspiro[4,4]non-3-en-2-one represent an important class of tetronic acids and part of them have high biological activity as herbicides and insecticides (Fischer *et al.*,  1995). Bayer company has developed three tetronic acids pesticides-spirodiclofen, spiromesifen and spirotetramat (Bayer Aktiengesellschaft, 1995). In addition, the title compound 3-mesityl-4-hydroxy-1-oxaspiro[4,4]non-3-en-2-one is the key intermediate in preparing highly efficient acaricide- spiromesifen. As part of our continuing interest in the new acaricide design and synthesis, We have isolated the product, (I), of the cyclized reaction of 1-(2-mesityl-acetoxy)-cyclopentanecarboxylic acid methyl ester as colorless crystals suitable for X-ray analysis.

The molecular structure of (I) is shown in Fig. 1. The molecule contains one benzene ring and two five membered rings. The dihedral angle between benzene and furan rings is 63.28 (15)°, smaller than the angle between benzene and furan rings of the compound 3-Mesityl-2-oxo-1-oxaspiro[4,4]non-3-en-4-yl-2-(4-chlorophenyl) -3-methylbutyrate (Yu *et al.*, 2009). The cyclopentyl ring displays an envelope conformation with C17 atom at the flap position 0.538 (3) Å out of the mean plane formed by the other four atoms. The title molecules are linked through an intermolecular hydrogen bond of O1—H1···O2. As expected, C2—C3 and C4—O2 are typically double bonds with bond distances of 1.344 (2) and 1.220 (2) Å. The bond distance of C3—C4 is 1.457 (2) Å, suggesting that carbonyl group on C4 has formed conjugate system with double bond on C3 and C2.

### Experimental

1-(2-Mesityl-acetoxy)-cyclopentanecarboxylic acid methyl ester (10 mmol, 3.04 g) was added to a solution of potassium t-butoxide (12 mmol, 1.34 g) in t-butylalcohol (35 ml) and the mixture was stirred at reflux for 5 h. Then water (70 ml) was added and the solution was acidified with hydrochloric (2*M*) to give a solid precipitate. The solid was filtrated and recrystallized with 95% ethanol to colourless blocks.

### Refinement

H atoms were included in calculated positions (C—H = 0.93–0.97 and O—H = 0.82 Å) and refined using a rinding model, with *U*_iso_(H) = 1.2*U*_eq_(C or O).

### Crystal data

**Table:**

C_17_H_20_O_3_	*F*(000) = 584
*M**_r_* = 272.33	*D*_x_ = 1.212 Mg m^−^^3^
Monoclinic, *P*2_1_/*n*	Mo *K*α radiation, λ = 0.71075 Å
Hall symbol: -P 2yn	Cell parameters from 9815 reflections
*a* = 8.8543 (4) Å	θ = 3.1–27.5°
*b* = 17.9266 (7) Å	µ = 0.08 mm^−^^1^
*c* = 9.4883 (4) Å	*T* = 296 K
β = 97.809 (2)°	Chunk, colorless
*V* = 1492.09 (11) Å^3^	0.54 × 0.48 × 0.20 mm
*Z* = 4	

### Data collection

**Table:**

Rigaku R-AXIS RAPID diffractometer	3410 independent reflections
Radiation source: fine-focus sealed tube	2344 reflections with *I* > 2σ(*I*)
graphite	*R*_int_ = 0.026
Detector resolution: 10.00 pixels mm^-1^	θ_max_ = 27.5°, θ_min_ = 3.1°
ω scans	*h* = −11→11
Absorption correction: multi-scan (*ABSCOR*; Higashi, 1995)	*k* = −22→23
*T*_min_ = 0.947, *T*_max_ = 0.984	*l* = −12→11
14502 measured reflections	

### Refinement

**Table:**

Refinement on *F*^2^	Secondary atom site location: difference Fourier map
Least-squares matrix: full	Hydrogen site location: inferred from neighbouring sites
*R*[*F*^2^ > 2σ(*F*^2^)] = 0.045	H-atom parameters constrained
*wR*(*F*^2^) = 0.139	*w* = 1/[σ^2^(*F*_o_^2^) + (0.0705*P*)^2^ + 0.2966*P*] where *P* = (*F*_o_^2^ + 2*F*_c_^2^)/3
*S* = 1.00	(Δ/σ)_max_ = 0.003
3410 reflections	Δρ_max_ = 0.21 e Å^−^^3^
186 parameters	Δρ_min_ = −0.17 e Å^−^^3^
0 restraints	Extinction correction: *SHELXL97* (Sheldrick, 2008), Fc^*^=kFc[1+0.001xFc^2^λ^3^/sin(2θ)]^-1/4^
Primary atom site location: structure-invariant direct methods	Extinction coefficient: 0.028 (4)

### Special details

**Table:**

Geometry. All e.s.d.'s (except the e.s.d. in the dihedral angle between two l.s. planes) are estimated using the full covariance matrix. The cell e.s.d.'s are taken into account individually in the estimation of e.s.d.'s in distances, angles and torsion angles; correlations between e.s.d.'s in cell parameters are only used when they are defined by crystal symmetry. An approximate (isotropic) treatment of cell e.s.d.'s is used for estimating e.s.d.'s involving l.s. planes.
Refinement. Refinement of *F*^2^ against ALL reflections. The weighted *R*-factor *wR* and goodness of fit *S* are based on *F*^2^, conventional *R*-factors *R* are based on *F*, with *F* set to zero for negative *F*^2^. The threshold expression of *F*^2^ > σ(*F*^2^) is used only for calculating *R*-factors(gt) *etc*. and is not relevant to the choice of reflections for refinement. *R*-factors based on *F*^2^ are statistically about twice as large as those based on *F*, and *R*- factors based on ALL data will be even larger.

### Fractional atomic coordinates and isotropic or equivalent isotropic displacement parameters (Å^2^)

**Table:**

	*x*	*y*	*z*	*U*_iso_*/*U*_eq_	
O1	0.58430 (13)	0.34534 (6)	0.74039 (12)	0.0565 (3)	
H1	0.6644	0.3224	0.7610	0.068*	
O2	0.35651 (13)	0.19282 (7)	0.36494 (12)	0.0566 (3)	
O3	0.28216 (11)	0.29401 (6)	0.47485 (11)	0.0509 (3)	
C1	0.34616 (17)	0.34474 (8)	0.58738 (16)	0.0479 (4)	
C2	0.49897 (16)	0.31085 (8)	0.63500 (15)	0.0441 (3)	
C3	0.52342 (15)	0.25023 (8)	0.55790 (15)	0.0423 (3)	
C4	0.38583 (16)	0.24044 (8)	0.45643 (16)	0.0449 (4)	
C5	0.66240 (15)	0.20313 (8)	0.56780 (14)	0.0418 (3)	
C6	0.65908 (17)	0.12818 (9)	0.60806 (15)	0.0466 (4)	
C7	0.7938 (2)	0.08727 (10)	0.62374 (18)	0.0577 (4)	
H7	0.7910	0.0373	0.6496	0.069*	
C8	0.93077 (19)	0.11821 (12)	0.60223 (19)	0.0630 (5)	
C9	0.93142 (18)	0.19158 (12)	0.55935 (19)	0.0632 (5)	
H9	1.0232	0.2129	0.5428	0.076*	
C10	0.79980 (17)	0.23510 (10)	0.53979 (17)	0.0516 (4)	
C11	0.5131 (2)	0.09125 (10)	0.6355 (2)	0.0669 (5)	
H11A	0.5348	0.0421	0.6729	0.080*	
H11B	0.4447	0.0879	0.5480	0.080*	
H11C	0.4665	0.1202	0.7029	0.080*	
C12	1.0770 (2)	0.07381 (16)	0.6244 (3)	0.0992 (9)	
H12A	1.1297	0.0795	0.5430	0.119*	
H12B	1.0539	0.0221	0.6365	0.119*	
H12C	1.1404	0.0916	0.7077	0.119*	
C13	0.8078 (3)	0.31327 (12)	0.4869 (3)	0.0788 (6)	
H13A	0.7232	0.3224	0.4144	0.095*	
H13B	0.9014	0.3202	0.4481	0.095*	
H13C	0.8039	0.3474	0.5642	0.095*	
C14	0.2387 (2)	0.35110 (11)	0.7003 (2)	0.0651 (5)	
H14A	0.1679	0.3095	0.6926	0.078*	
H14B	0.2964	0.3507	0.7948	0.078*	
C15	0.1532 (3)	0.42400 (14)	0.6740 (3)	0.0951 (8)	
H15A	0.0442	0.4157	0.6673	0.114*	
H15B	0.1836	0.4588	0.7509	0.114*	
C16	0.1930 (3)	0.45354 (13)	0.5383 (3)	0.0983 (9)	
H16A	0.1941	0.5076	0.5393	0.118*	
H16B	0.1201	0.4368	0.4590	0.118*	
C17	0.3512 (2)	0.42296 (10)	0.5263 (2)	0.0672 (5)	
H17A	0.3699	0.4215	0.4280	0.081*	
H17B	0.4297	0.4528	0.5811	0.081*	

### Atomic displacement parameters (Å^2^)

**Table:**

	*U*^11^	*U*^22^	*U*^33^	*U*^12^	*U*^13^	*U*^23^
O2	0.0467 (6)	0.0659 (7)	0.0516 (6)	0.0019 (5)	−0.0130 (5)	−0.0115 (5)
O3	0.0375 (5)	0.0604 (6)	0.0503 (6)	0.0066 (4)	−0.0107 (5)	−0.0036 (5)
O1	0.0467 (6)	0.0621 (7)	0.0544 (6)	0.0071 (5)	−0.0158 (5)	−0.0121 (5)
C6	0.0434 (8)	0.0533 (8)	0.0421 (7)	0.0022 (6)	0.0023 (6)	−0.0025 (6)
C2	0.0375 (7)	0.0511 (8)	0.0404 (7)	0.0005 (6)	−0.0062 (6)	0.0018 (6)
C5	0.0337 (7)	0.0544 (8)	0.0350 (7)	0.0002 (6)	−0.0033 (5)	−0.0035 (6)
C4	0.0362 (7)	0.0538 (8)	0.0420 (7)	−0.0003 (6)	−0.0040 (6)	0.0018 (7)
C7	0.0587 (10)	0.0606 (10)	0.0515 (9)	0.0148 (8)	−0.0014 (8)	−0.0052 (8)
C3	0.0331 (7)	0.0508 (8)	0.0403 (7)	−0.0003 (6)	−0.0045 (6)	0.0010 (6)
C1	0.0415 (8)	0.0530 (8)	0.0450 (8)	0.0063 (6)	−0.0086 (6)	−0.0003 (6)
C10	0.0393 (8)	0.0672 (10)	0.0471 (8)	−0.0077 (7)	0.0016 (6)	−0.0061 (7)
C9	0.0325 (8)	0.0966 (14)	0.0598 (10)	−0.0071 (8)	0.0035 (7)	−0.0207 (10)
C8	0.0434 (9)	0.0874 (13)	0.0544 (9)	0.0161 (9)	−0.0069 (7)	−0.0207 (9)
C11	0.0600 (11)	0.0624 (10)	0.0800 (12)	−0.0036 (8)	0.0157 (9)	0.0087 (9)
C14	0.0505 (10)	0.0856 (13)	0.0572 (10)	0.0117 (9)	0.0003 (8)	−0.0070 (9)
C17	0.0730 (12)	0.0551 (10)	0.0672 (11)	0.0017 (8)	−0.0126 (9)	0.0091 (8)
C13	0.0677 (13)	0.0812 (13)	0.0881 (14)	−0.0219 (10)	0.0133 (11)	0.0100 (11)
C16	0.0885 (17)	0.0668 (13)	0.127 (2)	0.0307 (12)	−0.0290 (16)	−0.0023 (14)
C12	0.0569 (12)	0.129 (2)	0.1046 (17)	0.0382 (13)	−0.0141 (12)	−0.0354 (16)
C15	0.0583 (12)	0.0889 (16)	0.135 (2)	0.0126 (11)	0.0030 (13)	−0.0317 (16)

### Geometric parameters (Å, °)

**Table:**

O2—C4	1.2200 (18)	C8—C12	1.510 (2)
O3—C4	1.3561 (18)	C11—H11A	0.9600
O3—C1	1.4574 (18)	C11—H11B	0.9600
O1—C2	1.3221 (17)	C11—H11C	0.9600
O1—H1	0.8200	C14—C15	1.514 (3)
C6—C7	1.391 (2)	C14—H14A	0.9700
C6—C5	1.398 (2)	C14—H14B	0.9700
C6—C11	1.506 (2)	C17—C16	1.523 (3)
C2—C3	1.344 (2)	C17—H17A	0.9700
C2—C1	1.4960 (19)	C17—H17B	0.9700
C5—C10	1.402 (2)	C13—H13A	0.9600
C5—C3	1.4847 (19)	C13—H13B	0.9600
C4—C3	1.4571 (19)	C13—H13C	0.9600
C7—C8	1.374 (3)	C16—C15	1.479 (4)
C7—H7	0.9300	C16—H16A	0.9700
C1—C17	1.520 (2)	C16—H16B	0.9700
C1—C14	1.531 (3)	C12—H12A	0.9600
C10—C9	1.394 (2)	C12—H12B	0.9600
C10—C13	1.493 (3)	C12—H12C	0.9600
C9—C8	1.377 (3)	C15—H15A	0.9700
C9—H9	0.9300	C15—H15B	0.9700
			
C4—O3—C1	109.49 (10)	H11A—C11—H11C	109.5
C2—O1—H1	109.5	H11B—C11—H11C	109.5
C7—C6—C5	119.10 (15)	C15—C14—C1	107.09 (18)
C7—C6—C11	119.57 (15)	C15—C14—H14A	110.3
C5—C6—C11	121.33 (14)	C1—C14—H14A	110.3
O1—C2—C3	132.24 (13)	C15—C14—H14B	110.3
O1—C2—C1	116.03 (13)	C1—C14—H14B	110.3
C3—C2—C1	111.72 (12)	H14A—C14—H14B	108.6
C6—C5—C10	119.70 (14)	C1—C17—C16	103.20 (18)
C6—C5—C3	121.04 (13)	C1—C17—H17A	111.1
C10—C5—C3	119.24 (14)	C16—C17—H17A	111.1
O2—C4—O3	120.32 (12)	C1—C17—H17B	111.1
O2—C4—C3	129.22 (14)	C16—C17—H17B	111.1
O3—C4—C3	110.45 (13)	H17A—C17—H17B	109.1
C8—C7—C6	122.20 (17)	C10—C13—H13A	109.5
C8—C7—H7	118.9	C10—C13—H13B	109.5
C6—C7—H7	118.9	H13A—C13—H13B	109.5
C2—C3—C4	105.96 (13)	C10—C13—H13C	109.5
C2—C3—C5	128.23 (12)	H13A—C13—H13C	109.5
C4—C3—C5	125.79 (13)	H13B—C13—H13C	109.5
O3—C1—C2	102.33 (11)	C15—C16—C17	105.47 (17)
O3—C1—C17	108.96 (12)	C15—C16—H16A	110.6
C2—C1—C17	114.67 (14)	C17—C16—H16A	110.6
O3—C1—C14	110.02 (13)	C15—C16—H16B	110.6
C2—C1—C14	116.21 (13)	C17—C16—H16B	110.6
C17—C1—C14	104.56 (14)	H16A—C16—H16B	108.8
C9—C10—C5	118.51 (16)	C8—C12—H12A	109.5
C9—C10—C13	119.54 (16)	C8—C12—H12B	109.5
C5—C10—C13	121.93 (16)	H12A—C12—H12B	109.5
C8—C9—C10	122.48 (16)	C8—C12—H12C	109.5
C8—C9—H9	118.8	H12A—C12—H12C	109.5
C10—C9—H9	118.8	H12B—C12—H12C	109.5
C7—C8—C9	117.95 (15)	C16—C15—C14	106.21 (19)
C7—C8—C12	121.6 (2)	C16—C15—H15A	110.5
C9—C8—C12	120.4 (2)	C14—C15—H15A	110.5
C6—C11—H11A	109.5	C16—C15—H15B	110.5
C6—C11—H11B	109.5	C14—C15—H15B	110.5
H11A—C11—H11B	109.5	H15A—C15—H15B	108.7
C6—C11—H11C	109.5		
			
C7—C6—C5—C10	1.9 (2)	C3—C2—C1—O3	1.92 (17)
C11—C6—C5—C10	−178.43 (15)	O1—C2—C1—C17	63.07 (19)
C7—C6—C5—C3	−176.40 (13)	C3—C2—C1—C17	−115.89 (16)
C11—C6—C5—C3	3.2 (2)	O1—C2—C1—C14	−59.24 (19)
C1—O3—C4—O2	−178.53 (14)	C3—C2—C1—C14	121.80 (16)
C1—O3—C4—C3	1.85 (17)	C6—C5—C10—C9	−2.9 (2)
C5—C6—C7—C8	0.6 (2)	C3—C5—C10—C9	175.51 (14)
C11—C6—C7—C8	−179.04 (16)	C6—C5—C10—C13	175.74 (16)
O1—C2—C3—C4	−179.64 (16)	C3—C5—C10—C13	−5.9 (2)
C1—C2—C3—C4	−0.90 (18)	C5—C10—C9—C8	1.3 (3)
O1—C2—C3—C5	−1.5 (3)	C13—C10—C9—C8	−177.29 (17)
C1—C2—C3—C5	177.20 (14)	C6—C7—C8—C9	−2.1 (2)
O2—C4—C3—C2	179.84 (16)	C6—C7—C8—C12	177.94 (17)
O3—C4—C3—C2	−0.58 (18)	C10—C9—C8—C7	1.1 (3)
O2—C4—C3—C5	1.7 (3)	C10—C9—C8—C12	−178.93 (18)
O3—C4—C3—C5	−178.75 (13)	O3—C1—C14—C15	−101.66 (17)
C6—C5—C3—C2	116.31 (18)	C2—C1—C14—C15	142.70 (16)
C10—C5—C3—C2	−62.0 (2)	C17—C1—C14—C15	15.21 (19)
C6—C5—C3—C4	−65.9 (2)	O3—C1—C17—C16	85.95 (18)
C10—C5—C3—C4	115.72 (17)	C2—C1—C17—C16	−160.07 (16)
C4—O3—C1—C2	−2.22 (16)	C14—C1—C17—C16	−31.65 (18)
C4—O3—C1—C17	119.58 (15)	C1—C17—C16—C15	37.3 (2)
C4—O3—C1—C14	−126.33 (14)	C17—C16—C15—C14	−27.9 (2)
O1—C2—C1—O3	−179.12 (13)	C1—C14—C15—C16	7.8 (2)

### Hydrogen-bond geometry (Å, °)

**Table:**

*D*—H···*A*	*D*—H	H···*A*	*D*···*A*	*D*—H···*A*
O1—H1···O2^i^	0.82	1.87	2.6267 (14)	154

Symmetry codes: (i) *x*+1/2, −*y*+1/2, *z*+1/2.
